# Prevalence and causes of low vision and blindness in an elderly population in Nepal: the Bhaktapur retina study

**DOI:** 10.1186/s12886-018-0710-9

**Published:** 2018-02-13

**Authors:** Raba Thapa, Sanyam Bajimaya, Govinda Paudyal, Shankar Khanal, Stevie Tan, Suman S. Thapa, G. H. M. B. van Rens

**Affiliations:** 1 Vitreo-retina Service, Tilganga Institute of Ophthalmology, P O Box 561, Kathmandu, Nepal; 2 Central Department of Statistics, Tribhuvan University, Kirtipur, Nepal; 3 Vrije University Medical Center, Amsterdam, The Netherlands

**Keywords:** Blindness, Elderly, Low vision, Nepal, Refractive error, Prevalence

## Abstract

**Background:**

This study aims to explore the prevalence and causes of low vision and blindness focused on retinal disease in a population above 60 years in Nepal.

**Methods:**

Two thousand one hundred subjects were enrolled in a population-based cross-sectional study. History, presenting and best corrected visual acuity after subjective refraction, anterior and posterior segment examinations was obtained in detail.

**Results:**

Among the total subjects, 1860 (88.57%) had complete information. Age varies from 60 to 95 (mean age: 69.64 ± 7.31) years. Low vision and blindness in both eyes at presentation was found in 984 (52.90%, 95% confidence interval (CI): 50.60–55.19) and 36 (1.94%, 95% CI: 1.35–2.66) subjects respectively. After best correction, bilateral low vision and blindness was found in 426 (22.92%, 95% CI: 21.01–24.88), and 30 (1.61%, 95% CI: 0.10–2.30) subjects respectively. As compared to 60–69 years old, risk of visual impairment was four times higher (95% CI:3.26–5.58) in the 70–79 year olds and 14 times higher (95% CI: 9.72–19.73) in the age group 80 years and above.

Major causes of bilateral low vision were cataract (68.07%), followed by retinal disorders (28.64%), and for blindness; retinal disorders (46.66%), followed by cataract (43.33%). Illiteracy was significantly associated with visual impairment.

**Conclusion:**

Among the elderly population, prevalence of visual impairment was high. Refractive error, cataract and retinal disorders were the major cause of low vision. Screening the population at the age 60 years and above, focused on cataract and posterior segment diseases, providing glasses and timely referral can help reduce visual impairment.

## Background

Low vision and blindness are serious global public health problems with increasing prevalence due to shifting of demographics and aging populations [1, 2]. A recent systematic review and meta- analysis by the Vision Loss Expert Group estimated that 36 million people to be blind and 216.6 million people to have moderate to severe visual impairment globally [3].

More than 80% of the global visual impairment burden is preventable, and more than 90% of the visually impaired people live in developing countries [4]. Besides the aging population, the prevalence of visual impairment is on the rise in developing countries due in part to the low level of health care infrastructure [5]. Cataract is the most common cause of blindness in the developing world [6–12], while retinal disorders are the commonest cause of blindness in the developed world [13–19]. Uncorrected refractive error however remains to be a major cause of visual impairment too [1, 4, 7, 13, 15, 20].

In the past decades, not much changed in the causes of visual impairment; however the relative prevalence of cataract is decreasing. Cataract (39% and 33%), uncorrected refractive error (20% and 21%) and macular degeneration (5% and 7%) were reported as the most important causes of blindness in 1990 and 2010 respectively. Similarly, uncorrected refractive error (51% and 53%), cataract (26% and 18%) and macular degeneration (2% and 3%) were the most common causes of moderate and severe visual impairment in 1990 and 2010 [21].

The first study on the prevalence of visual impairment in Nepal, the Nepal Blindness Survey (NBS), conducted in 1981, estimated the overall prevalence of bilateral blindness at 0.84%. The prevalence was 3.8% in the age group of 45 years and above. Cataract was the most common cause (83%) of blindness in this age group, and more than 80% of this overall blindness was either curable or treatable at all age groups [22]. Several other population-based studies of Nepal have reported the prevalence of blindness to range from 2.0–5.3%. Cataract was the predominant cause of blindness in all studies [23–25].

The Bhaktapur Glaucoma Study (BGS) conducted 5 years ago, reported a low prevalence of visual impairment in the 40–60 years age group (0.93%) [26]. Therefore, the follow-up study present here included subjects above the age of 60 years only. This Bhaktapur Retina Study (BRS) aims to assess the population prevalence and causes of visual impairment among the age group of 60 years and above in the Bhaktapur district in Nepal.

## Methods

### Study population

During the study period, Bhaktapur district comprised of 2 municipalities and 161 village development committees. The population of Bhaktapur was 298,704 and 48,223 were above the age of 40 years as reported by National population census 2001. The details of the study methods have been published in a companion paper [27, 28]. In brief, the required sample size for this BRS was estimated to be 2100 subjects after assuming 7% prevalence for retinal disorders in individuals 60 years and older, a relative precision of 25%, 85% compliance, and a design effect of 2.The 7% prevalence of retinal disorders was derived from the occurrence of retinal disorders in the BGS [26]. The study sample comprised of the BGS sample conducted from 2007 to 2010, where a WHO 30 cluster sampling method was used. A house to house enumeration was carried out and a name list prepared from these selected 30 clusters. From this name list 4800 subjects above the age of 40 years were selected using EPI-INFO software, version 3.5.1 [29]. In BRS, only those subjects of 60 years of age and above were re-invited for an eye-examination. 62% of the original samples above the age of 60 years of the BGS still were alive during the BRS; from the remaining subjects, 18% of the subjects were unable to visit the study site, 15% had passed away and 5% had moved to other places. The rest (38%) study subjects were selected from the adjoining clusters as a cross sectional survey to meet the required sample size of 2100. Two female community health workers were involved to invite the study subjects in this study. The prevalence and causes of low vision (visual acuity of less than 6/18 (< 20/60; < 0.3 Log MAR) but not less than 3/60 (20/400; 1.3 Log MAR) in the better eye after best correction) and blindness (Visual acuity of less than 3/60 (< 20/200; < 1.3 Log MAR) with best correction in the better eye) were assessed in this population. All subjects underwent a detailed ocular examination at the community eye centre in the Bhaktapur district. The study subjects were enrolled from August 2013 to December 2015 in the BRS.

A structured questionnaire was developed to assess the prevalence and risk factors for visual impairment. The interview was conducted by the mid-level ophthalmic personnel where as eye examination was conducted by the two ophthalmologists. Pre-testing was done in 50 cases. None of the respondents reported difficulties in answering the questionnaire, and no statistically significant variations were identified in examination findings.

### Patient examination, low vision, and blindness assessments

Detailed ocular and medical history was taken from all study subjects, followed by anterior segment, dilated fundus examinations and intraocular pressure measurement. Logarithm of minimum angle of resolution (logMAR) with tumbling E charts placed at 4 m (4 m original series ETDRS Chart; company: Precision Vision) was used to assess visual acuity**.** The uncorrected and best corrected visual acuities (BCVA) were recorded. Streak retinoscopy (Beta 200, Heine, Germany) was used for objective refraction, and this was followed by subjective refraction. If the subjects were not able to read the logMAR 1.0 line, the vision was again checked at 1 m. If the subjects were unable to recognize any of the largest optotypes, then perception of hand movement was checked. If hand movement also not recognized, then presence of light perception was checked and recorded in the proforma. Two retina specialists were involved in **s**tandardized eye examinations on the patients.

International Statistical Classification of Disease 10th revision was used for visual impairment definition [30]. Briefly, visual impairment was considered when visual acuity (VA) of less than 6/18 (< 20/60; < 0.3 LogMAR) in the better eye with best correction. Low vision was considered when a BCVA of less than 6/18 (< 20/60; < 0.3 LogMAR) but not less than 3/60 (20/400; 1.3 LogMAR) in the better eye. A VA of less than 3/60 (< 20/200; < 1.3 LogMAR) with best correction in the better eye was defined as blindness. Uncorrected refractive error was defined as refractive error that had not been corrected in the past or that which was inadequately corrected [31]. Cataract was graded according to Lens Opacities Classification System III (LOCS III) [32]. Diabetic retinopathy was graded using Early Treatment Diabetic Retinopathy Study (ETDRS) criteria [33]. Briefly, DR was graded as non proliferative diabetic retinopathy (NPDR) and proliferative diabetic retinopathy (PDR). Subjects were categorized having any retinopathy if they had any form of NPDR or PDR at least in one eye. Similarly, all the subjects with DR have been included irrespective of the stages of DR in this study.

Age-related macular degeneration (AMD) was graded according to the International classification developed by the International age-related maculopathy (ARM) Epidemiological Study Group by one of the retina specialist at each clinical examination [34]. Briefly, ARM is a degenerative disorder in persons ≥50 years of age having the following abnormalities in the macula: soft drusen ≥63 μm, hyperpigmentation and /or hypopigmentation of the retinal pigment epithelium (RPE), RPE and associated neurosensory detachment, (peri) retinal hemorrhage, geographic atrophy of the RPE, or (peri) retinal fibrous scarring in the absence of other retinal (vascular) disorders. All the stages of AMD were included in this study.

Hypertensive retinopathy was graded according to Modified Scheie Classification as Grade 0; no changes, grade 1; barely detectable arterial narrowing, grade 2; obvious arterial narrowing with focal irregularities, grade 3; grade two plus retinal hemorrhage and or exudates, grade 4; grade three plus disc swelling [35].

Firstly, all causes in each eye that contributed to vision loss alone were identified. If any disease was a secondary cause from other pathology, the primary cause was selected as the principal cause. When two causes were identified in one eye, a cause that was treatable and where the treatment would, in the ophthalmologist’s opinion, improve the vision was given precedence over a cause that was untreatable. If a cause was preventable but not treatable, it was identified as the primary cause if the other causes for the eye were neither treatable nor preventable. If a cause was neither treatable nor preventable, the ophthalmologists used their clinical judgment to identify the principal cause for the eye. Treatable and preventable causes were therefore preferentially selected over unavoidable causes. The main cause in the right eye or left eye was chosen thereafter to represent the principal cause for the person. If the causes in right and left eye differed, the principal cause for the person was selected as the one more amenable to treatment, or, if not treatable, more amenable to prevention [36].

### Definitions and assessment of risk factors

A standardized questionnaire was used to take the history. By using standard techniques, blood examination for non-fasting blood sugar levels, measurements of blood pressure, height, weight, and abdominal girth were recorded from all subjects. By self reported history taking, age, gender, literacy, occupation, and presence of systemic problems like diabetes mellitus, hypertension was included.

Literates were categorized for those subjects who were able to read and write in the national language as defined by the Government of Nepal.

Those involved in the farming were categorized belonging to the agriculture occupation. Office goers, business, health professionals etc. were grouped under other occupations.

The diagnosis of diabetes mellitus was done based upon the use of hypoglycaemic medications or a non-fasting blood sugar level in the venous blood sample of 200 mg/dl or greater [29, 37]. Blood pressure (BP) was measured on all participants. The study participants were categorized as hypertensive if they were under antihypertensive medications or if the systolic blood pressure was 140 mmHg or above, diastolic blood pressure was 90 mmHg or above.

The Institutional Review Board and Ethics Committee (IRC) of Tilganga Institute of Ophthalmology (TIO) had approved the study (reference number of ethics committee approval: 01/2013/IRC-TIO). The study was conducted in accordance with the Declaration of Helsinki. Informed consent was written in the vernacular and was read out for the illiterates. Written informed consent was taken and thumb impressions were taken from the illiterates prior to enrollment in the study.

### Statistical analysis

Descriptive statistical measures such as mean ± Standard Deviation (SD) for continuous variables and percentages were computed for categorical variables. Association between two independent categorical variables was assessed by using Chi-square or Fisher’s exact test wherever applicable. The effects of different independent variables on visual impairment after best correction were examined by univariate and multiple logistic regression analysis. All the results were considered significant if *p*-value < 0.05 at 5% level of significance. Statistical analysis was performed using STATA 13.0, College Station, Texas, USA.

## Results

A total of 1860 (88.57%) subjects were considered with complete information in this study. There was no significant difference found between the non-responders and responders groups in the age and gender (Table 1).

**Table 1:** Distribution of responders and non responders in the study population

Variable	Responders (*N* = 1860)	Non responders (*N* = 240)	*p*- value
Mean age (Years)	69.64 ± 7.31	69.50 ± 7.93	0.782
Male, N (%)	821 (44.14)	110 (45.83)	0.629
Female, N (%)	1039 (55.86)	130 (54.17)	

*Abbreviation*: *N* Number

Table 2 explains the demographic characteristics of the study population. The age of all the study subjects varies from 60 to 95 years with the mean age of 69.64 ± 7.31 years. The mean age was almost similar between men and women (men 69.98 ± 7.37 SD and women 69.36 ± 7.26 SD). The study subjects between 60 and 69 years of age comprised of 51.08% while the subjects 80 years and above were 11.45%. Among the total study subjects, females were 1039 (55.86%), 1433 (77.04%) were illiterate and 1351 (72.63%) were farmers by occupation.

**Table 2:** Demographic characteristics of the study population

Characteristics	All Subjects (*N* = 1860)	
	Frequency	Percent
Age (years):		
60–69	950	51.08
70–79	697	37.47
≥ 80	213	11.45
Gender:		
Men	821	44.14
Women	1039	55.86
Occupation:		
Agricultural	1351	72.63
Others	509	27.37
Literacy:		
Illiteracy	1433	77.04
Literacy	427	22.96

*Abbreviation*: *N* Number

Table 3 shows the presenting and best corrected visual acuity among the different groups. Among the total subjects, 840 (45.16%, 95% Confidence Interval (CI): 42.88–47.46) had no visual impairment, 984 (52.90%, 95% CI: 50.60–55.19) had low vision, and 36 (1.94%, 95% CI: 1.35–2.66) were blind at the time of presentation. The visual acuity was worse with increasing age (*p* <  0.001). After best correction, 1404 (75.48%, 95% CI: 73.46–77.42) had no visual impairment, 426 (22.92%, 95% CI: 21.01–24.88) had low vision, and 30 (1.61%, 95% CI: 0.10–2.30) were blind. In this group, the visual acuity was similarly worse with increasing age (*p* <  0.001). Gender was not associated with visual impairment both in the presenting visual acuity (*p* = 0.153) and best corrected visual acuity group (*p* = 0.314). Visual impairment among the BCVA group was found to be significantly higher among the subjects involved in agricultural occupations (*p* = 0.001). Similarly, visual impairment was found to be significantly higher among the persons who were illiterate (*p* <  0.001) in both the presenting and BCVA group.

**Table 3:** Presenting visual acuity and best corrected visual acuity in better eye

	Presenting visual acuity in better eye (N/%)			*P* value	Best Corrected visual acuity in better eye (N/%)			*P* value
	No visual impairment	Low vision	Blindness		No visual impairment	Low vision	Blindness	
Age (years):								
60–69	555(58.42)	390(41.05)	5(0.53)	< 0.001¥	856(90.11)	90(9.47)	4(0.42)	< 0.001¥
70–79	253(36.30)	428(61.41)	16(2.30)		466(66.86)	220(31.56)	11(1.58)	
≥ 80	32(15.02)	166(77.93)	15(7.04)	< 0.001¥	82(38.50)	116(54.50)	15(7.04)	< 0.001¥
Gender:								
Men	386(47.02)	412(50.18)	23(2.80)	0.153ǂ	629(76.61)	171(20.83)	21(2.56)	0.314ǂ
Women	454(43.70)	572(55.05)	13(1.25)		775(74.59)	255(24.54)	9(0.87)	
Occupation:								
Agricultural	606(44.86)	717(53.07)	28(2.07)	0.666ǂ	993(73.45)	335(24.80)	24(1.78)	0.001ǂ
Others	234(45.97)	267(52.46)	8(1.57)		411(80.91)	91(17.91)	6(1.18)	
Literacy:								
Illiterates	588(41.03)	813(56.73)	32(2.23)	< 0.001ǂ	1033(72.08)	373(26.03)	27(1.88)	< 0.001ǂ
Literates	252(59.02)	171(40.05)	4(0.94)		371(86.89)	53(12.41)	3(0.70)	
Total	840(45.16)	984(52.90)	36(1.94)		1404(75.48)	426(22.92)	30(1.61)	

No Visual impairment (< 0.3 LogMAR), Low vision (< 0.3 LogMAR ≥1.3 LogMAR), Blindness (> 1.3 LogMAR)

*Abbreviation*: *N* Number

¥p-value is comparing the proportion of visual impairment in age group 70–79 years and 80 years and above each with 60–69 years using Chi-square test

ǂ *p*-value is comparing the proportion of visual impairment across gender, literacy and occupation each using Chi-square test. Presenting visual acuity and best corrected visual acuity (BCVA) measured in LogMAR

Among the four predictors considered in this study, age (*p* <  0.001), occupation (*p* = 0.001) and literacy (*p* <  0.001) were significantly associated with the visual impairment in univariate analysis, whereas gender was not. Only two variables, age (*p* <  0.001) and literacy (*p* <  0.001), were found significantly associated with visual impairment in multivariate analysis. The risk of developing visual impairment in the70–79 years age group was four times higher (95% CI:3.26–5.58) and 14 times higher (95% CI; 9.72–19.73) in the age group 80 years and more as compared with the 60–69 years age group (Table 4).

**Table 4:** Effect of age, gender, occupation and literacy on visual impairment after best correction in better eye using Logistic regression model

Variable	No visual impairment (%) (*N* = 1404)	Visual impairment (%) (*N* = 456)	Univariate analysis		Multivariable analysis	
			OR(95% CI)	P value	OR(95% CI)	*P* value
Age(years)						
60–69	856(60.97)	94 (20.61)	1		1	
70–79	466(33.19)	231(50.66)	4.51(3.46–5.88)	< 0.001	4.27(3.26–5.58)	< 0.001
≥ 80	82(5.84)	131 (28.73)	14.72(10.38–20.88)	< 0.001	13.85(9.72–19.73)	< 0.001
Gender:						
Men	629(44.80)	192 (42.11)	1		1	
Women	775(55.19)	264 (57.89)	1.11(0.90–1.38)	0.308	1.04(0.80–1.35)	0.733
Occupation:						
Agricultural	992(70.65)	359 (78.73)	1		1	
Others	412(29.34)	97 (21.27)	0.65(0.50–0.83)	0.001	0.77(0.58–1.03)	0.083
Literacy:						
Illiteracy	1033(73.57)	400 (87.72)	1		1	
Literacy	371(26.42)	56 (12.28)	0.38(0.28–0.52)	< 0.001	0.52(0.36–0.75)	< 0.001

Best corrected visual acuity (BCVA) in better eye measured in LogMAR. No Visual impairment (< 0.3 logMAR), visual impairment (both low vision (< 0.3 LogMAR≥1.3 LogMAR), and blindness (> 1.3 LogMAR)

All the independent variables for visual impairment after best correction considered in the univariate analysis were included for analysis in multivariable logistic regression model

*Abbreviation*: *N* Number, *OR* Odds Ratio, *CI* Confidence Interval

Among the total 426 subjects of bilateral low vision, cataract in 290 (68.07%) people, followed by retinal disorders in 122 (28.64%) people, corneal scar in four (5.63%) people, glaucoma and posterior capsular opacity in three (0.70%) people each were the major causes of low vision after best correction in better eye.

Among the subjects with unilateral low vision, cataract was again the predominant cause in 364 (76.15%) people followed by retinal disorders in 100 (20.92%) people, corneal scar 5 (1.04%) people, glaucoma and posterior capsular opacification (PCO) in four (0.84%) people each. Similarly, retinal disorders in 14 (46.66%) people and cataract in 13 (43.33%) people were the major causes of bilateral blindness. Cataract in 65(55.08%) people, retinal disorders in 38 (32.20%) people, phthisis bulbi in five (4.25%) people, corneal scar in three (2.54%) people, primary glaucoma in two (1.69%) people, secondary glaucoma in two (1.69%) people, and amblyopia in two (1.69%) people were the major causes of unilateral blindness (Table 5).

**Table 5:** Causes of visual impairment in better eye after best correction

Cause	Unilateral low vision (Number/Percent)	Bilateral low vision (Number/Percent)	Unilateral blindness (Number/Percent)	Bilateral blindness (Number/Percent)
Cataract	364 (76.15)	290 (68.07)	65(55.08)	13 (43.33)
Retinal disorders	100 (20.92)	122 (28.64)	38 (32.20)	14(46.66)
Corneal scar	5 (1.04)	4(5.63)	3 (2.54)	1(3.33)
Glaucoma	4 (0.84)	3 (0.70)	2 (1.69)	
Optic atrophy		2 (0.47)		1 (3.33)
PCO	4 (0.84)	3 (0.70)		1 (3.33)
Asteroid hyalosis	1(0.20)	2 (0.47)		
Secondary glaucoma			2 (1.69)	
Phthisis bulbi			5 (4.24)	
Amblyopia			2 (1.69)	
Advanced pterygium			1 (0.85)	
Total	478 (25.69% of all)	426 (22.9% of all)	118 (6.34% of all)	30 (1.61% of all)

Best corrected visual acuity (BCVA) in better eye measured in LogMAR

Low vision (< 0.3 LogMAR ≥1.3 LogMAR), Blindness (> 1.3 LogMAR)

*Abbreviation*: *PCO* Posterior Sub capsular Cataract

Among the 100 people with unilateral low vision due to retinal diseases, dry AMD in 56 (56%) cases, BRVO and diabetic retinopathy each in 10 (10%), epi-retinal membrane (ERM) in eight (8%), myopic retinal degeneration and macular scar each representing in 4 (4%) cases were identified.

Similarly, among the 122 people of bilateral low vision due to retinal causes, dry AMD was the most common cause in 64 (52.45%) cases followed by branch retinal vein occlusion (BRVO) in 13 (10.65%), ERM in five (9.01%), DR in six (5.75%), myopic retinal degeneration in seven (5.74%), macular hole in five (4.09%), macular scar in three (3.28%) and wet AMD in three (2.46%) cases were identified. Among the 38 subjects with unilateral blindness due to retinal causes; dry AMD in seven (18.42%) cases, wet AMD in six (15.79%), ERM in six (15.79%), myopic retinal degeneration in five (13.16%), DR in three (7.89%), BRVO in two (5.26%), macular scar in three (7.29%), and macular hole in two (5.26%) cases were identified. Similarly, among the 14 subjects with bilateral blindness due to retinal causes, dry AMD in six (42.85%) cases, wet AMD in five (35.71%), myopic retinal degeneration in two (14.28%), and retinal detachment in one (7.14%) cases were identified. None of the subjects had grade four hypertensive retinopathy in the study (Table 6).

**Table 6:** Retinal causes of visual impairment in better eye after best correction

Cause	Unilateral low vision	Bilateral low vision	Unilateral blindness	Bilateral blindness
Dry AMD	56 (56)	64 (52.45)	7 (18.42)	6 (42.85)
Wet AMD	2 (2)	3 (2.46)	6 (15.79)	5 (35.71)
BRVO	10 (10)	13 (10.65)	2 (5.26)	
ERM	8 (8)	11 (9.01)	6 (15.79)	
CRVO	2 (2)	2 (1.64)	1 (2.63)	
Diabetic retinopathy	10 (10)	7 (5.74)	3 (7.89)	
Macular hole	1 (1)	5 (4.09)	2 (5.26)	
Myopic fundus	4 (4)	7 (5.74)	5 (13.16)	2 (14.28)
Macular scar	4 (4)	4 (3.28)	3 (7.89)	
Retinitis pigmentosa		1 (0.82)		
Retinal detachment		1 (0.82)	1 (2.63)	1 (7.14)
Hypertensive retinopathy (grade three)	2 (2)	3 (2.46)	1 (2.63)	
Undetermined cause		1 (0.82)	1 (2.63)	
Chorio-retinal coloboma	1 (1)			
Total	100	122	38	14

Best corrected visual acuity (BCVA) in better eye measured in LogMAR

Low vision (< 0.3 LogMAR ≥1.3 LogMAR), Blindness (> 1.3 LogMAR)

*Abbreviation*: *AMD* Age Related Macular Degeneration, *BRVO* Branch Retinal Vein Occlusion, *CRVO* Central Retinal Vein Occlusion, *ERM* Epi Retinal Membrane

## Discussion

The Bhaktapur district is an adjoining district of Kathmandu, the capital city of Nepal. It has no tertiary eye hospital but is served by two primary eye care centers. The majority of the population is able to access the tertiary eye hospitals of Kathmandu in average of 90 min and average distance of 25 km. In this study, we present the prevalence of low vision and blindness in the Bhaktapur district in Nepal among inhabitants older than 60 years of age.

The mean age of the study participants was 69.64 ± 7.31 years, ranging from 60 to 95 years. There was a predominance of females (55.86%), illiterates (77.04%), and farmers (72.63%).

Based on presenting visual acuity, the prevalence of low vision and blindness was 52.90% and 1.94%, respectively. After best correction, the prevalence of low vision and blindness was 22.92% and 1.61%, respectively. Presenting visual acuity improved by 29.98% of subjects with low vision and 0.33% of subjects with blindness after the use of spectacles. This showed that uncorrected refractive error was a major cause contributing to low vision and blindness. Routine vision screening, regular follow up after cataract surgery and provision of spectacles have to be emphasized in this community to reduce these rates of avoidable visual impairment due to uncorrected refractive error. This finding was consistent with other studies both from the developed and developing world [4, 7, 13, 15, 20, 38–41].

The prevalence of blindness at presentation was 1.94% in our study. The low prevalence of blindness in our study is partly due to reduction of cataract blindness and improved visual outcomes from modern cataract surgery. A higher utilization of service could have lead to improved screening and treatment of ocular diseases.

In the BGS, the prevalence of blindness after best correction was 0.48% in the age group 60–69 years, 1.89% in the age group 70–79 years, and 2.06% in the age group above 80 years [20], whereas in our study, the prevalence of blindness was 0.42% in the age group 60–69 years, 1.58% in the age group 70–79 years old, and 7.08% at the age group 80 years and above. A higher rate of blindness at the age group 80 years and above could be due to an increased participation of subjects from this age group (213; 11.45% of study population) in our study compared to the BGS (97; 2.42% of the study population). The BRS was conducted in the district whereas the BGS was conducted at the base hospital. Therefore, this could have also resulted in a higher participation among the very elderly age group.

In the NBS conducted in 1981among the blind population [22], the prevalence of blindness was 61.8% at the age group 60 years and above. There has been a dramatic reduction in the rates of blindness since 1981. The reduction in the prevalence of blindness has been mainly due to the improvement in the eye health care facilities especially due to an increase in the cataract surgical coverage (CSC). The CSC for the visual acuity < 3/60 was 35% in the 1981 NBS [22], 58% in the 1995 survey [24], 85% in an nationwide RAAB survey conducted in 2010 [25], while the CSC of Bhaktapur district in 2006 was reported 90% in BGS [20], Also, the implantation of intraocular lenses in cataract surgery has resulted in better visual outcomes. This can be reflected by the Lumbini eye study conducted in 1995 and 2006 where visual outcomes of cataract surgery had improved over the past 10 years [23, 24]. Among the subjects who had undergone cataract surgery, the presenting visual acuity of more than or equal to 6/18 in Bhaktapur district was 54.5% in 2006, while the Lumbini survey in 2006 reported 61.4%, an enormous improvement compared to the Lumbini survey 1995 result that reported just 15% [20, 24, 38]. Only 15% cases undergoing cataract surgery in 1995 had IOL implantation while almost 90% of cases had IOL implantation in 2006 [24, 38]. The low rate of blindness in the present study reflected the acceptability for the cataract surgery.

In this study, cataracts (68.07%) followed by retinal disorders (28.64%) were the major causes of low vision after best correction. In BGS, cataract and retinal disorders was the first and second most common cause of blindness responsible for 60.8% and 11.4% respectively [20]. Similarly, cataracts (43.33%) and retinal disorders (46.66%) were again the major causes of bilateral blindness in the BRS while in the BGS, cataract and retinal diseases comprising of 47.1% and 14.7% respectively. These similar findings to the BGS [20] which was conducted in the same population six years ago, reiterates that cataract still remains the major cause of blindness. Thirty years ago the NBS figures found that cataract was responsible for 66.8% and retinal disorder 3.3% of bilateral blindness. Thus blindness caused by retinal disorders in this study was higher when compared to both the BGS and NBS [20, 21]. This could probably for a part be due to an inclusion of a more elderly age group, increase in life expectancy and also an increase in age related retinal disorders, especially those related to systemic hypertension and diabetes. However, this also could for a part be due to a better coverage of cataract service leading to increase in proportion of other diseases. Among the retinal causes for low vision and blindness, AMD was the most common cause, followed by retinal vascular diseases such as RVO and DR. Our findings suggest that with the aging population, retinal diseases become major causes for blindness. Our findings are consistent with other studies [4, 6, 13–15, 17, 19]. A recommendation of healthy lifestyle, which includes healthy diet, cessation of smoking and the use of protective measures for sunlight exposure, can help prevent AMD as well as diabetes and so DR [42–45]. Increased utilization of allied ophthalmic personnel trained in fundus photography for early screening of retinal diseases and subsequent referral of vision threatening conditions such as DR could help reduce blindness in resource-limited developing countries [46]. The burden of retinal blindness will become a serious public health problem in the coming years with this increasing trend in Nepal as well as globally. Timely precautions and improved treatment facilities are recommended to address this problem [4, 6, 12, 14–16, 18, 23–25].

The rates of blindness increased with age. The higher prevalence of cataract and increase in the frequency of age related ocular disorders could be the possible reasons for the higher rate of blindness in this elderly age group of 80 years and above. Our findings agree with other studies conducted in the region [20, 26, 47, 48] as well as around the world [3, 4, 12].

Age (*p* <  0.001) and illiteracy (p <  0.001) were found to be significantly associated with visual impairment in multivariate analyses. The risk of developing visual impairment in the 70–79 years age group was four times higher (95% CI:3.26–5.58) and 14 times higher (95% CI; 9.72–19.73) in the age group 80 years and more, as compared with the 60–69 year age group. The increase in visual impairment with increase in age was consistent with other studies from Nepal and neighboring countries [20, 21, 26, 47, 48]. The possible reason for this is due to increase in age related eye diseases especially retinal diseases, which are not treatable, such as advanced dry AMD.

Females had a slightly higher prevalence of visual impairment; however this was not statistically significant. Our finding is consistent with the report from the BGS [20] and other studies conducted in Nepal [22, 24]. None of the studies in Nepal reported visual impairment higher in males as compared to females. The possible reason for more visual impairment among females could be due to unequal access to healthcare and social stigma in wearing spectacles. While analyzing for cataract as the cause of low vision and blindness, females had a higher prevalence of both unilateral (53.13%) and bilateral low vision (59.31%). However, there were also more females with pseudophakia in one eye (55.22%) and both eyes (58.39%).This shows that females had benefitted the most by utilizing cataract surgery services. In a recent National RAAB survey conducted in 2010, there was also no significant difference for cataract surgical coverage among the gender [25]. The major barrier for the cataract surgery was found to be the inability to afford surgery [25].

Visual impairment among the BCVA group was found to be significantly higher among the subjects involved in agricultural occupations (*p* = 0.001) and among the persons who were illiterate in both the presenting and BCVA group (*p* <  0.001). These findings were similar to the Pakistan National Blindness survey [48]. As AMD was the major cause of blindness, we can state that probably protective measures to prevent sunlight exposure were not taken by those working in the field. Similarly, this disparity could also probably be due to a higher level of awareness among the literates and other occupational groups [49] who could have had access to better quality of food, less exposure to sunlight, increased use of protective sunglasses, and timely eye checkups.

The majority of the causes of low vision and blindness found in our study can be treated. Despite this fact, our findings suggest that awareness campaigns and treatment strategies against major blinding diseases should be focused on those at the age 60 years and above to prevent or treat low vision and blindness.

The strength of the study is the large sample size of the elderly population and the detailed retinal examination carried out. The weakness is that our study comprised only one district in Nepal.

## Conclusion

Prevalence of visual impairment in Nepal was high among the age group 60 years and above. Refractive error was the major cause of low vision, followed by cataract and retinal disorders. Screening of those populations at the age 60 years and above, providing glasses and timely referral and treatment can help reduce visual impairment.

## Abbreviations

AMD: Age Related Macular Degeneration
ARM: Age Related Maculopathy
BCVA: Best Corrected Visual Acuity
BGS: Bhaktapur Glaucoma Study
BP: Blood Pressure
BRS: Bhaktapur Retina Study
BRVO: Branch Retinal Vein Occlusion
CI: Confidence Interval
CRVO: Central Retinal Vein Occlusion
CSC: Cataract Surgical Coverage
DR: Diabetic Retinopathy
ERM: Epi Retinal Membrane
ETDRS: Early Treatment Diabetic Retinopathy Study
LOCS: Lens Opacities Classification System
LogMAR: Logathirm of Minimum Angle of Resolution
N: Number
NBS: Nepal Blindness Survey
PCO: Posterior Capsular Opacification
RAAB: Rapid Assessment of Avoidable Blindness
SD: Standard Deviation
TIO: Tilganga Institute of Ophthalmology
WHO: World Health Organization
